# Triterpenoid CDDO-EA Protects from Hyperglycemia, Hyperinsulinemia, and Obesity by Decreasing Energy Intake

**DOI:** 10.3390/ijms26125485

**Published:** 2025-06-07

**Authors:** Austin E. Cantu, Cordelia Rasa, Shizue Mito, Denae Cantu, Juan Carlos Lopez-Alvarenga, Leslie L. Rivera-Lopez, Israel Rios, Ashley Abrego-Gonzalez, Sara M. Reyna

**Affiliations:** 1 Baylor College of Medicine, Houston, TX 76798, USA; austin.cantu@bcm.edu; 2 Department of Laboratory Animal Resources, The University of Texas Rio Grande Valley, Edinburg, TX 78539, USA; 3 Department of Medical Education, School of Medicine, The University of Texas Rio Grande Valley, Edinburg, TX 78541, USA; 4 School of Podiatric Medicine, The University of Texas Rio Grande Valley, Edinburg, TX 78539, USA; 5 Division of Population Health and Biostatistics, School of Medicine, The University of Texas Rio Grande Valley, Edinburg, TX 78539, USA; juan.lopezalvarenga@utrgv.edu; 6 Department of Translational Sciences, Graduate School of Biomedical Sciences, The University of Texas Health Science Center at San Antonio, 7703 Floyd Curl Drive, San Antonio, TX 78229, USA; 7 Division of Human Genetics, School of Medicine, The University of Texas Rio Grande Valley, Edinburg, TX 78539, USA

**Keywords:** CDDO-EA, obesity, insulin resistance, type 2 diabetes, mouse model

## Abstract

Obesity is a significant factor in the development of type 2 diabetes (T2D). Treatment of obesity is pivotal in the prevention and management of T2D, and the development of new pharmacological therapies are studied for improving insulin resistance and glucose intolerance. Oleanolic acid-derived triterpenoids, 2-cyano-3,12-dioxoolean-1,9-dien-28-oic acids (CDDOs), are studied to elucidate the mechanisms by which they protect against obesity. However, fundamental knowledge gaps remain regarding the physiological and molecular mechanisms by which CDDOs protect against obesity. Our recently published studies showed that CDDO-ethyl amide (CDDO-EA) prevents skeletal muscle inflammation by inhibiting activation of nuclear factor-kappa B (NF-κB) signaling. Moreover, CDDO-EA induced translocation of glucose transporter 4, GLUT4, in skeletal muscle cells. We hypothesized that CDDO-EA protects from obesity-induced hyperglycemia in mice fed a high-fat diet (HFD). Our results show that CDDO-EA protects from HFD-induced obesity but has no effect on body weight in mice fed a low-fat diet (LFD). Our data show that CDDO-EA inhibition of weight gain is associated with reduced caloric intake and glucose and insulin levels in mice fed an HFD. This highlights the potential of CDDO-EA as a therapeutic agent for obesity treatment and the protection against the development of T2D.

## 1. Introduction

Obesity is a significant risk factor associated with the development and progression to T2D in which hyperglycemia is the primary manifestation [1]. Furthermore, obesity is the fastest-growing pandemic in the world, and it is estimated that 738 million individuals will be diagnosed with T2D by 2045 [2]. Obese individuals develop insulin resistance, which is characterized by impaired insulin sensitivity by tissues [1], leading to hyperglycemia and hyperinsulinemia, which can lead to serious complications such as neuropathies, cancer, and cardiovascular disorders [3,4,5]. Therefore, the treatment and prevention of obesity, insulin resistance, and T2D are important to prevent these serious complications.

Oleanolic acid-derived triterpenoids exhibit anti-inflammatory, anti-tumorigenic, and anti-diabetic properties [6]. In particular, the CDDO derivatives CDDO-methyl ester (CDDO-Me) and CDDO-imidazole (CDDO-Im) have been studied for their anti-diabetic properties [7,8,9,10]. Recently, we demonstrated that CDDO-EA protects skeletal muscle cells from lipopolysaccharide (LPS)-induced inflammation by inhibiting NF-κB activation [11]. Our work also showed that CDDO-EA induced translocation of GLUT4 in skeletal muscle cells. Our findings show that CDDO-EA protects from weight gain by preventing energy intake. Also, we show that the prevention of obesity by CDDO-EA is associated with a reduction in hyperglycemia and hyperinsulinemia. Our findings suggest that CDDO-EA holds potential as a future therapeutic agent against obesity to protect from the development of insulin resistance and T2D.

## 2. Results

### 2.1. Validation of CDDO-EA Synthesis

**Figure 1:**
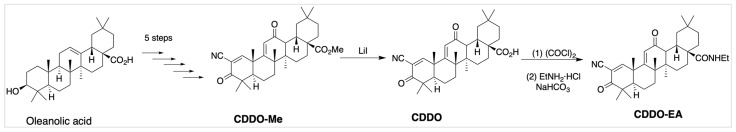
Synthesis of CDDO-EA.

CDDO-Me was prepared from oleanolic acid by following the reported procedure (Figure 1) [12]. CDDO-Me was then hydrolyzed to CDDO, and the subsequent amide coupling gave CDDO-EA. The structure of synthesized CDDO-EA was confirmed with ^1^H nuclear magnetic resonance (NMR) and high-resolution mass spectrometry (HRMS), and the purity was verified by high-performance liquid chromatography (HPLC) (>99%) (see Supplemental Materials).

### 2.2. CDDO-EA Suppresses LPS-Induced MCP-1 Production in Macrophages

**Figure 2:**
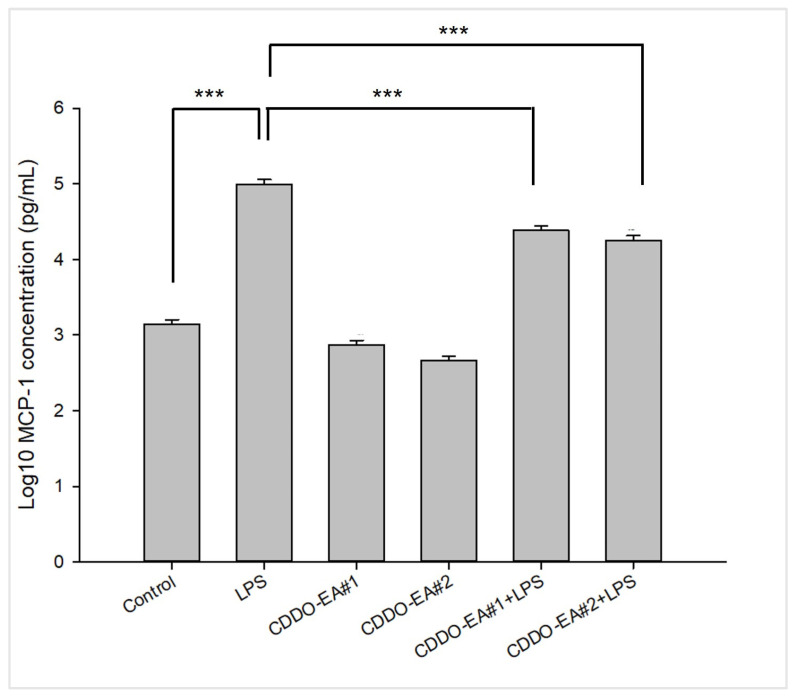
Synthesized CDDO-EA suppresses MCP-1 production in macrophages. RAW264.7 mouse macrophages were pre-treated with 500 nM CDDO-EA for 1 h then exposed to 100 ng/mL LPS for 6 h. Data represented as mean ± SEM. *** *p* < 0.001 (one-way ANOVA).

Our synthesized CDDO-EA was tested for its biological property of anti-inflammatory activity. As with the original CDDO-EA, which we used in our previous study and was a kind gift from Dr. Thomas Slaga from the University of Texas Health Science Center at San Antonio [11], we tested two different batches of CDDO-EA synthesized by us for their biological activity to suppress monocyte chemotactic protein-1 (MCP-1) production in macrophages. As shown in Figure 2, both batches of CDDO-EA significantly decreased LPS-induced production of MCP-1 in RAW264.7 macrophages. This suppression of MCP-1 production shows that our synthesized CDDO-EA has anti-inflammatory properties like the original CDDO-EA [11].

### 2.3. CDDO-EA Protects from High-Fat Diet-Induced Obesity

**Figure 3:**
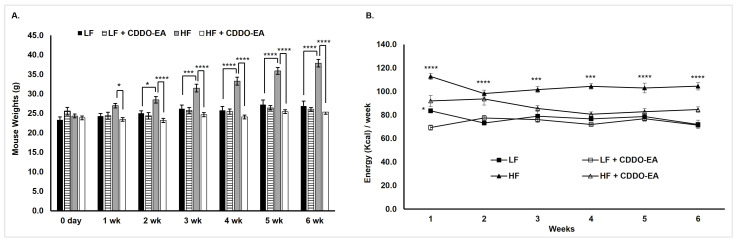
CDDO-EA prevents weight gain by inhibiting energy intake. C57Bl6/J mice were fed an LFD (*n* = 5) or HFD (*n* = 7) without CDDO-EA or LFD (*n* = 7) or HFD (*n* = 7) with incorporation of CDDO-EA (diet containing 0.04% CDDO-EA) for 6 weeks. Data are represented as mean ± SEM; two-way repeated measures ANOVA. (**A**) * *p* < 0.05, *** *p* < 0.001, **** *p* < 0.0001. (**B**) * *p* < 0.05 vs. LFD + CDDO-EA, *** *p* < 0.001 vs. HFD + CDDO-EA, **** *p* < 0.0001 vs. HFD + CDDO-EA.

As shown in Figure 3A, our findings show that mice fed an HFD weighed significantly more than the LFD-fed mice by week two, and this was consistent throughout the six-week study. The incorporation of CDDO-EA in the HFD prevented excess weight gain compared to an HFD alone. We then measured energy intake and found that CDDO-EA decreased energy intake in mice fed an HFD (Figure 3B). Mice fed an LFD with CDDO-EA show a decrease in energy intake only in week one (Figure 3B); however, this effect could be due to adaptation and acclimation of mice to a new environment and feed [13,14]. Importantly, this effect did not persist throughout the duration of this study, and CDDO-EA did not affect body weight in mice fed an LFD (Figure 3A).

### 2.4. CDDO-EA Prevents Hyperglycemia and Hyperinsulinemia

**Figure 4:**
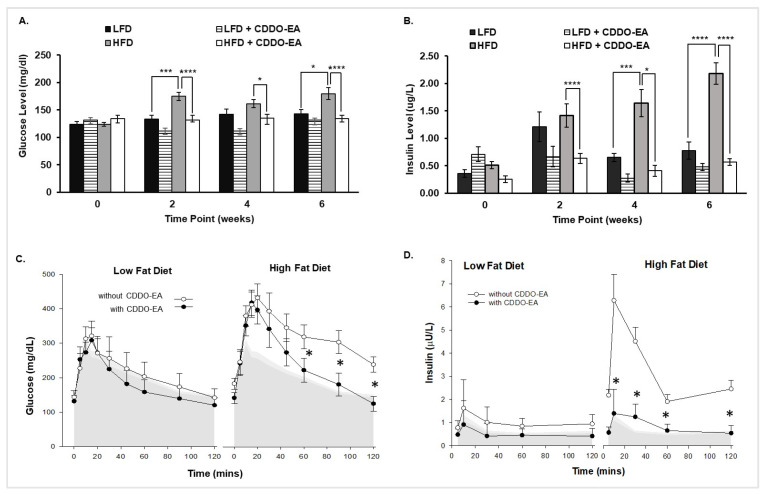
CDDO-EA prevents glucose intolerance and hyperinsulinemia. C57Bl6/J mice were fed an LFD or HFD with or without incorporation of CDDO-EA (diet containing 0.04% CDDO-EA) for 6 weeks. LFD, *n* = 5; LFD + CDDO-EA, *n* = 7; HFD, *n* = 7; HFD + CDDO-EA, *n* = 7. (**A**,**B**) Data are represented as mean ± SEM; two-way repeated measures ANOVA. * *p* < 0.05, *** *p* < 0.001, **** *p* < 0.0001. (**C**,**D**) Blood glucose (**A**) and plasma insulin (**B**) measurements obtained from OGTTs. The left panel of each graph corresponds to LFD and the right panel of each graph corresponds to HFD. ANOVA: Differences by fat diet at basal *p* = 0.89, final *p* < 0.001. Differences by CDDO-EA at basal *p* = 0.64, final *p* = 0.003. The gray shaded area represents the mean values of glucose (**A**) or insulin (**B**) concentration (basal levels) before experimental feeding. The error bars correspond to 95% confidence intervals.

We further measured glucose and insulin levels to determine if prevention of obesity by CDDO-EA coincided with a reduction in hyperglycemia and hyperinsulinemia. Blood glucose levels significantly increased in mice fed an HFD at 2 weeks and remained significantly increased throughout the rest of the period, as shown in Figure 4A. In contrast, blood glucose levels in mice fed an HFD with CDDO-EA did not increase throughout this six-week study. In addition, Figure 4B shows that plasma insulin levels were significantly higher at 2 weeks and remained significantly higher in mice fed only an HFD compared to mice fed an HFD with CDDO-EA. The LFD group had increased plasma insulin levels compared with the LFD + CDDO-EA group but without statistical significance. The increased insulin levels can reflect normal metabolic adaptation in the absence of dietary stressors like a HFD. For example, there were significant differences in insulin levels between LFD and HFD in weeks 4 and 6. As seen in Figure 4C,D, oral glucose tolerance test (OGTT) showed that CDDO-EA prevented increased blood glucose and plasma insulin concentrations in mice fed an HFD. In Figure 4C, the gray shaded area corresponds to the blood glucose levels before experimental feeding. The glucose levels of both the LFD and LFD + CDDO-EA groups (left panel Figure 4C) overlap with the glucose levels before experimental feeding. Further, the HFD group’s glucose levels (right panel Figure 4C) are significantly higher after the 6-week HFD feeding. Interestingly, the HFD + CDDO-EA group’s glucose levels overlap with the HFD up to the 45 min timepoint and then significantly decrease to glucose levels before experimental feeding. In Figure 4D, the gray shaded area corresponds to the insulin levels before experimental feeding. The insulin levels of LFD and LFD + CDDO-EA after the 6-week experimental feeding overlap with the insulin levels before experimental feeding (left panel Figure 4D). In addition, the insulin levels of the HFD group (right panel Figure 4D) are significantly higher after the 6-week HFD feeding. The HFD + CDDO-EA group insulin levels did not increase significantly and overlap with the insulin levels before experimental feeding. Our findings show that CDDO-EA did not affect glucose and insulin levels of mice fed an LFD with CDDO-EA, and HFD feeding alone induces glucose intolerance and hyperinsulinemia. However, CDDO-EA incorporation in the HFD inhibited increases in glucose and insulin levels.

## 3. Discussion

Obesity is a high-risk factor for the development of insulin resistance, and one of the main causes of insulin resistance is impaired glucose transport in tissues, like in the skeletal muscle [1]. Skeletal muscle plays a crucial role in whole body glucose metabolism, and GLUT4 is the primary glucose transporter in skeletal muscle. Although many studies have examined anti-diabetic properties of CDDOs to protect from obesity and T2D, more studies are needed to examine their role in protecting from obesity-induced insulin resistance. This led us to study whether CDDO-EA induces GLUT4 translocation, and we previously showed that CDDO-EA indeed induced the translocation of GLUT4 in skeletal muscle cells [11].

In the present study, we evaluated whether CDDO-EA could protect from obesity-induced insulin resistance in an animal model. Our findings demonstrate that CDDO-EA protects from obesity-induced insulin resistance by inhibiting weight gain due to reduced food intake and improving insulin sensitivity and glucose metabolism. We previously confirmed NF-kB inhibition and GLUT4 translocation in vitro may explain the observed in vivo effects of CDDO-EA [8].

CDDO derivatives are known nuclear factor erythroid 2-related factor 2 (Nrf2) activators, regulating anti-oxidant and anti-inflammatory genes and improving mitochondrial function [15]. It is possible that the protective effects of CDDOs can involve muscle, adipose tissue, and liver through the Nrf2 pathway. However, these activated pathways by CDDOs crosstalk with other molecules and may have more actions than anti-oxidant effects [16]. A possible action by which CDDO-EA regulates food intake is by influencing hypothalamic appetite regulation. Still, crossing the blood–brain barrier remains to be further studied as well as whether reductions in hypothalamic inflammation and oxidative stress by CDDOs affect thermoregulation or diet-induced leptin resistance [17]. The potential interaction with gut microbiota should be considered, as recent studies have shown that notoginsenoside, a bioactive compound in plants, can modulate signaling pathways related to CDDOs [18]. Also, other triterpenoids modify gut microbiota composition, which have cerebral protection and improve metabolic outcomes in mice [19]. CDDO-EA shows similar benefits to glucagon-like peptide 1 (GLP-1) receptor agonists. Multi-modal benefits include reducing body weight, improving insulin sensitivity, and potentially modulating appetite. However, unlike GLP-1 agonists, which act primarily via the central nervous system and pancreas [20], CDDO-EA may exert systemic anti-inflammatory effects, offering a broader therapeutic scope.

Figure 4B showed longitudinal effect of CDDO-EA on insulin levels. There were differences in insulin concentration among LDF and HFD groups that may reflect compensatory hyperinsulinemia in response to a continued HFD stress. Since CDDOs are Nrf2 activators [15], CDDO-EA could activate Nrf2 upregulating anti-oxidant enzymes [21]. Thus, CDDO-EA exposure could involve an improvement on beta cell function, perhaps for a protective role on pancreatic cells from oxidative stress. NF- kB signaling and MCP-1 play critical roles in the development of insulin resistance [22,23], and we have shown that CDDO-EA suppresses NF-kB signaling and expression of pro-inflammatory cytokines and chemokines in skeletal muscle cells [11]. Also, our previously published work [11] and Figure 2 show that CDDO-EA significantly reduces MCP-1 production in macrophages. These could also be protective mechanisms by which CDDO-EA improves insulin sensitivity in peripheral tissues, such as skeletal muscle, liver, adipose tissue, and pancreas [6].

CDDO-Me and CDDO-Im have been mainly studied for their anti-obesity and anti-diabetic properties, and our findings add another CDDO derivative, which has similar properties with some important differences. Shin et al. conducted a four-day indirect calorimetry study in C57BL/6J female mice after 82 days of HFD (60% calories from fat) feeding and an oral gavage of CDDO-Im three times a week [7]. During the indirect calorimetry study, mice were dosed with CDDO-Im on day 0 and 2. This resulted in an acute and significant decrease in food intake only on day 0. Unlike in Shin et al., where food intake was not measured during the 82-day experimental feeding, our study measured food intake once a week for 6 weeks. Hence, our findings demonstrate a long-term food intake reduction and prevention in weight gain in mice fed an HFD feed incorporated with CDDO-EA.

Furthermore, it was not determined whether CDDO-Im prevented elevated blood glucose and plasma insulin levels in animals fed an HFD for 82 days [7]. We show that CDDO-EA protects from HFD-induced elevated blood glucose and plasma insulin levels. Our study also shows that these effects were not seen in mice fed an LFD into which CDDO-EA had been incorporated, demonstrating that CDDO-EA effects are specific to HFD feeding.

Contradictory findings have been reported for CDDO-Me on its anti-obesity and anti-diabetic properties. Camer et al. fed C57BL/6J male mice an HFD (40% calories from fat) for 12–16 weeks and then orally gavaged them with CDDO-Me for two weeks. Although these mice showed lower fasting blood glucose and plasma insulin levels, they had similar food intake and body weight to the control obese mice fed an HFD and treated with vehicle [8]. In other published reports, C57Bl6/J male mice were fed an HFD (40% calories from fat) and given an oral dose of CDDO-Me in drinking water for 21 weeks [9,10]. In the reported study, CDDO-Me reduced body weight in mice fed an HFD. Although not consistent throughout the 21-week study, a reduction in energy intake was also observed during different weeks of the study in mice given CDDO-Me. Although these mice showed reduced body weight gain and food intake, CDDO-Me normalized glucose levels at 120 min in a glucose tolerance test (GTT) following intraperitoneal injection of glucose. During the OGTT we performed, mice were orally gavaged with glucose, and we show that CDDO-EA normalized glucose levels starting at 60 min. Further, CDDO-Me lowered insulin levels in mice fed an HFD compared to an HFD alone. This result is similar to our study in that CDDO-EA prevented an increase in fasting insulin levels in mice fed an HFD. The discrepancies in the animal studies could be due to the differences in the CDDO derivatives studied, method and duration of CDDO derivative administration, dose of CDDO derivative, percent of fat in diets, and sex of animals.

Our study has some limitations, such as the exclusive use of male mice. Sex hormones have an effect on metabolism [24]. Future research should include female mice to analyze sex specific efficacy. Another pending study includes understanding the long-term efficacy and safety of CDDO-EA beyond 6 weeks. CDDO-Me (Bardoxolone) was evaluated in human trials for chronic kidney disease and T2D and found to have more frequent adverse events compared with the placebo group [25]. Studies to evaluate pharmacologic and toxicological effects of CDDO-EA are needed before considering clinical applications.

Our present study demonstrates that incorporation of CDDO-EA in an HFD prevents development of insulin resistance and improves glucose homeostasis by inhibiting energy intake and obesity. We will need further mechanistic studies of CDDO-EA on central and peripheral metabolic pathways. Future studies are needed to evaluate whether CDDO-EA acts through hypothalamic signaling and if this alters the inflammatory profile and its interaction with the gut–liver–muscle–brain axis. Evaluating the in vivo effects of CDDO-EA on GLUT4 expression, AMPK activity, and Nrf2-regulated proteins will increase the understanding of the mechanisms. Finally, a multi-omics approach will elucidate with major precision the therapeutic development of CDDO derivatives.

## 4. Materials and Methods

### 4.1. Animals and Diets

All procedures were approved by the University of Texas Rio Grande Valley Institutional Animal Care and Use Committee (Protocol # 17-05, approved 28 August 2018). Male C57BL/6J mice (6–8 weeks old, strain # 000664) were purchased from the Jackson Laboratory (Bar Harbor, ME, USA). Mice were fed irradiated rodent diets *ad libitum* as follows: LFD (10% of total calories from fat, #TD.08806, Envigo, Indianapolis, IN, USA) or an HFD (60% of total calories from fat, #TD.06414, Envigo, Indianapolis, IN, USA) without or with CDDO-EA (diet containing 0.04% CDDO-EA, which is 400 mg of CDDO-EA/kg of diet, and prepared by Envigo (Indianapolis, IN, USA) from CDDO-EA provided by our laboratory) for six weeks. The CDDO-EA concentration was based on reports showing that CDDO-EA incorporation in the feed is well tolerated by mice without affecting palatability and body weight resulting in protection from oxidative stress and inflammation in mouse models of Huntington’s disease and Lou Gehring’s disease [26,27]. Number of mice per treatment group: LFD, *n* = 5; LFD + CDDO-EA, *n* = 7; HFD, *n* = 7; and HFD + CDDO-EA, *n* = 7. Mice were individually housed under controlled temperature (23 °C) and lighting (10 h ligh:14 h dark) with free access to water and weekly, pre-measured feed. Feed consumption was calculated per cage by subtracting the remaining feed from the weight of feed provided at the beginning of the week. Both mice and any remaining rodent diet in each cage were weighed once a week. Energy intake in Kcal was calculated by multiplying the Kcal/g of low-fat diet (3.6 Kcal/g) with or without CDDO-EA or high-fat diet (5.1 Kcal/g) with or without CDDO-EA by the grams of weekly food consumption.

### 4.2. Synthesis of CDDO-EA

For our animal study, we synthesized CDDO-EA from oleanolic acid (Figure 1). First, CDDO-Me was prepared from oleanolic acid by the following the reported procedures [12]. Then, CDDO-Me was hydrolyzed in the presence of lithium iodide in dimethylformamide to produce CDDO [28]. Next, CDDO was treated with oxalyl chloride to form acid chloride, which was immediately mixed with ethylamine hydrochloride to complete the synthesis of CDDO-EA. The reaction mixture was purified by column chromatography. Oleanolic acid was purchased from Accela Chem Bio Inc (San Diego, CA, USA). Other chemical reagents were purchased from Sigma Aldrich (St. Louis, MO, USA), Alfa Aesar (Ward Hill, MA, USA), Acros Organic (Geel, BE), Fisher Scientific (Pittsburgh, PA, USA), and Thermo Scientific (Waltham, MA, USA). Chemicals and were used without further purification. All solvents were purchased from Fischer Scientific (Pittsburgh, PA, USA) and used without further purification. Flash column chromatography was performed on Thermo Scientific (Waltham, MA, USA) Siliga gel 60 (0.035–0.070 mm). The structure of the products was confirmed after each step by ^1^H NMR. The structure of CDDO-EA was confirmed with ^1^H NMR and HRMS, and the purity was verified by HPLC (Figures S1–S6).

### 4.3. RAW264.7 Cells

Mouse RAW 264.7 macrophage cells were cultured as described previously [29]. The cells were sub-cultured by using 5ml of ice-cold 5mM ethylediamineteraacetic acid (EDTA, catalog # E8008-100ML, Sigma Aldrich, St. Louis, MO, USA) in phosphate-buffered saline (PBS, catalog # 10010023, Gibco, Waltham, MA, USA) at 4 °C for 20 min, with the flask being tapped every 5 min to detach cells. The cell pellet was collected by centrifugation at 800 rpm for 5 min at room temperature.

### 4.4. MCP-1 Detection

RAW 264.7 cells were grown on 24-well dishes at 1 × 10^6^ cells/mL, using 0.5 mL/well. Concentration of CDDO-EA and duration of CDDO-EA pre-treatment was performed as previously described [11]. Cells were pre-treated with 500 nM CDDO-EA for 1 h. After 1 h, cells were treated with 100 ng/mL of LPS (*Escherichia coli* O111:B4, catalog # L5293, St. Louis, MO, USA) for 6 h, and supernatants were collected and stored at −80 °C until analysis. Treatments were run in triplicate. MCP-1 from the culture media of cells was measured and quantified using a Mouse MCP-1 ELISA Set (catalog # 555260, BD Biosciences, Milpitas, CA, USA) according to the manufacturer’s instructions. Samples were run in duplicate in two independent MCP-1 ELISAs.

### 4.5. Oral Glucose Tolerance Tests and Glucose Measurements

Oral glucose tolerance tests were performed before the start and at week 6 of the experimental feeding. The mice were fasted for 5 h. During OGTT, mice were awake and kept in their home cages between timepoints. A solution of 20% dextrose (2 g dextrose/kg) was given to each mouse via oral gavage (22 G, stainless steel, curved, 1.5″, Braintree Scientific, Braintree, MA, USA). The 20% dextrose solution was prepared from a 50% dextrose solution (catalog # 059384, Henry Schein Animal Health LLC, Miami, FL, USA) mixed with sterile milli-Q water using aseptic techniques. Blood samples were serially collected after a one-time removal of 1–2 mm off the tips of the tail with a razor blade, and blood glucose levels were measured by using a glucometer (Bayer Contour, Mishawaka, IN, USA). Blood glucose levels were measured at −5 min before glucose gavage and at 5, 10, 15, 20, 30, 45, 60, 90, and 120 min after glucose gavage. Blood samples were collected via tail tips for insulin measurements at −5 min before glucose gavage and at 10, 30, 60, and 120 min after glucose gavage. Blood samples for insulin measurement were collected in blood collection tubes coated with heparin and then centrifuged for 1 min at 13,000 rpm to collect plasma. Plasma samples were then carefully pipetted to a microcentrifuge tube and stored at −80 °C until ready for use. Blood glucose levels measured at −5 min for OGTT were used for 0- and 6-week timepoints. Blood samples were also collected via tail tips at 2 and 4 weeks into the experimental feeding period for unfasted blood glucose measurements using a glucometer (Bayer Contour, Mishawaka, IN, USA).

### 4.6. Insulin ELISA

Blood samples for measurement of insulin were collected during the OGTTs (before and at 6-week experimental feeding) and at 2 and 4 weeks into the experimental feeding. At 2 and 4 weeks, measurement of insulin levels were taken without fasting. Insulin was analyzed with a Mercodia (Winston-Salem, NC, USA) mouse insulin ELISA (catalog # 10-1247-01) with one well per serum sample. ELISA was performed according to the manufacturer’s instructions.

### 4.7. Statistical Analysis

All values were presented as mean ± SEM. Data were statistically analyzed by one-way ANOVA or two-way ANOVA with repeated measures. *p*-values equal to or less than 0.05 were considered significant. * *p* < 0.05; ** *p* < 0.01; *** *p* < 0.001; **** *p* < 0.0001.

## Data Availability

The raw data supporting the conclusions of this article will be made available by the authors on request.

## Supplementary Materials

The following supporting information can be downloaded at: https://www.mdpi.com/article/10.3390/ijms26125485/s1. Reference [30] is cited in the Supplementary Materials.

## Abbreviations

The following abbreviations are used in this manuscript:

CDDO	2-cyano-3,12-dioxoolean-1,9-dien-28-oic acid
CDDO-EA	2-cyano-3,12-dioxoolean-1,9-dien-28-oic acid-ethyl amide
CDDO-Cl	2-cyano-3,12-dioxoolean-1,9-dien-28-oic acid-chloride
CDDO-Im	2-cyano-3,12-dioxoolean-1,9-dien-28-oic acid-imidazole
CDDO-Me	2-cyano-3,12-dioxoolean-1,9-dien-28-oic acid-methyl ester
EDTA	ethylenediaminetetraacetic acid
ELISA	enzyme-linked immunosorbent assay
GLP-1	glucagon-like peptide 1
GLUT4	glucose transporter 4
GTT	glucose tolerance test
HFD	high-fat diet
HRMS	high-resolution mass spectrometry
HPLC	high-performance liquid chromatography
LFD	low-fat diet
MCP-1	monocyte chemotactic protein-1
MeOH	methanol
NF-κB	nuclear factor-kappa B
Nrf2	nuclear factor erythroid 2-related factor 2
NMR	nuclear magnetic resonance
OGTT	oral glucose tolerance test
PBS	phosphate-buffered saline
T2D	type 2 diabetes
