# Supplementary material for: Triterpenoid CDDO-EA Protects from Hyperglycemia, Hyperinsulinemia, and Obesity by Decreasing Energy Intake
Source: Int J Mol Sci. 2025 Jun 7;26(12):5485. doi: 10.3390/ijms26125485 (PMC12193413; doi:10.3390/ijms26125485)
Supplement: Supplementary file 1 [file ijms-26-05485-s001.zip › ijms-3624897-supplementary.pdf]

Supplemental Material

Triterpenoid CDDO-EA Protects from Hyperglycemia, Hyperinsulinemia, and Obesity by Decreasing Energy Intake

The synthesis of CDDO-EA

CDDO-Me was synthesized by reported procedure [1]

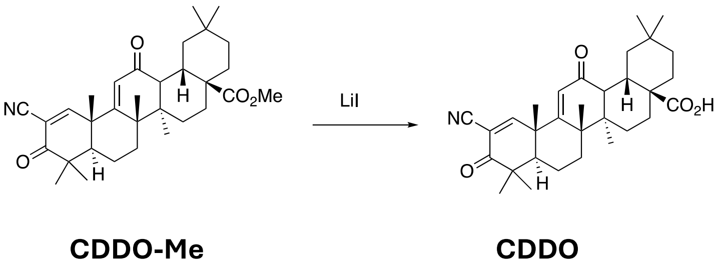

Figure S1: Synthesis of bardoxolone (CDDO) [2]

CDDO-Me (800 mg, 1.58 mmol, 1 eq) and dry LiI (3.9 g, 29.3 mmol, 18.5 eq) in dry DMF (15 mL) was heated under reflux for 4 h. The solution is quenched with 5% HCl aq. The mixture was extracted with EtOAc three times. The organic extract was washed with water three times, sat. NaCl aq., and dried over Na_2SO_4. The solvent was evaporated. Purification by column chromatography (dichloromethane only to dichloromethane: methanol = 90:10) to give 478 mg (62%) of CDDO

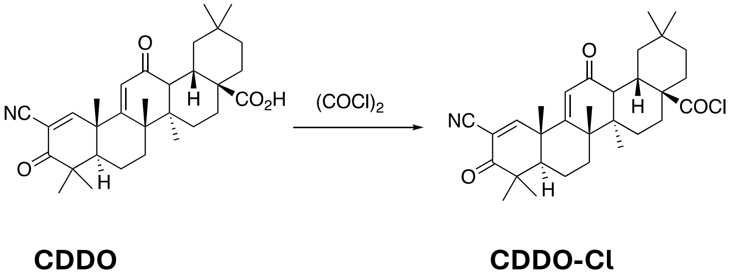

Figure S2: Synthesis of CDDO-Cl [3]

A mixture of CDDO (769 mg, 1.56 mmol, 1.0 eq) and oxalyl chloride (2.24 g, 1.51 mL, 17.7 mmol, 11.3 eq) in anhydrous dichloromethane (5 mL) was stirred at room temperature overnight. The solvent was evaporated, and the residue was co-evaporated with benzene three times. 786 mg (99%) of crude CDDO-Cl was obtained. This was used for the next step without further purification.

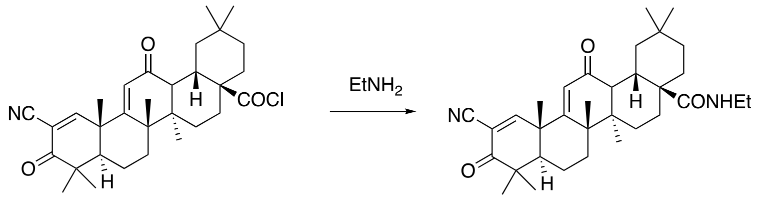

CDDO-Cl **CDDO-EA**

Figure S3: Synthesis of CDDO-EA [3]

The solution of CDDO-Cl (786 mg, 1.54 mmol, 1.0 eq) in benzene (15 mL) was added to the solution of ethylamine hydrochloride (285 mg, 3.89 mmol, 2.2 eq) and $NaHCO_3$ (690 mg, 8.47 mmol, 5.5 eq) in water (15 mL). The mixture was stirred at room temperature overnight. The layers were separated, and the aqueous layer was extracted with benzene (15 mL). The combined organic layers were washed with sat. $NaHCO_3$ aq, water, and sat. NaCl aq, and dried over Na_2SO_4. The solvent was evaporated. Purification by column chromatography (dichloromethane only to dichloromethane: methanol = 90:10) to give 521 mg (65%) of CDDO-EA. To get pure compound, the repeated column chromatography was required. The compound purity was confirmed by HPLC and NMR (> 99 %).

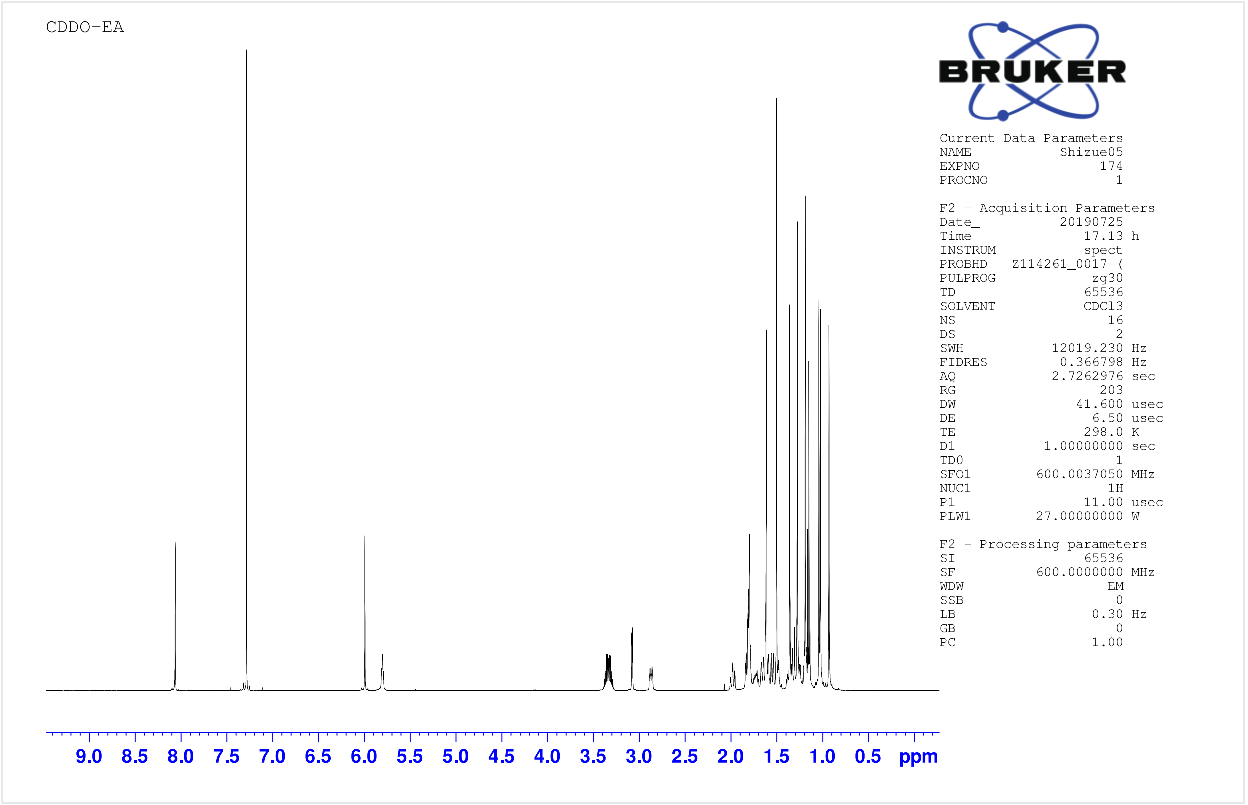

Figure S4. ^{1}H NMR of CDDO-EA.

^{1}H NMR spectrum was recorded on a Bruker 600 MHz spectrometer and chemical shifts are referenced to internal solvent resonances; multiplicities are indicated by s (singlet), d (doublet), t (triplet), q (quartet), m (multiplet) and br (broad). Coupling constants, *J*, are reported in Hertz. ^{1}H NMR (600 MHz, CDCl3) δ 8.06 (s, 1H), 5.99 (s, 1H), 5.80 (t, 1H, *J* = 5.4), 3.27-3.40 (m, 2H), 3.08 (d, 1H, *J* = 4.5 Hz), 2.87 (d, 1H, *J* = 12.8 Hz), 1.99 (td, 1H, *J* = 13.8, 3.8 Hz), 1.77-1.85 (m, 5H), 1.68-1.76 (m, 1H) 1.52-1.68 (m, 6H), 1.50 (s, 3H), 1.35 (s, 3H), 1.28 (s, 3H), 1.30-1.39 (m, 2H) 1.19 (s, 3H), 1.15 (t, 3H, *J* =7.2 Hz), 1.04 (s, 3H), 1.02 (s, 3H), 0.93 (s, 3H);

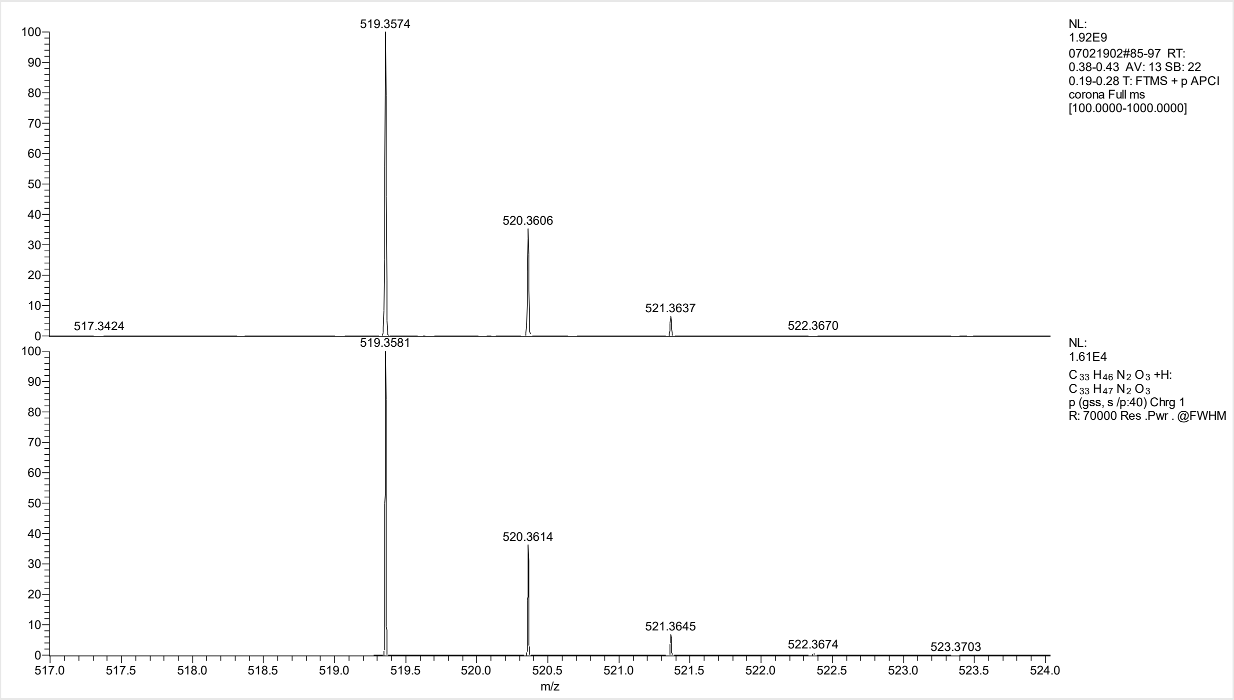

Figure S5. Mass spectrum of CDDO-EA (top) and calculated data (bottom).

HRMS (calcd. for C33H47N2O3 $[M+H]^{+}$) 519.3581, found 519.3574.

Mass spectrum is obtained by Thermo Scientific QE Focus with atmospheric pressure chemical ionization (APCI).

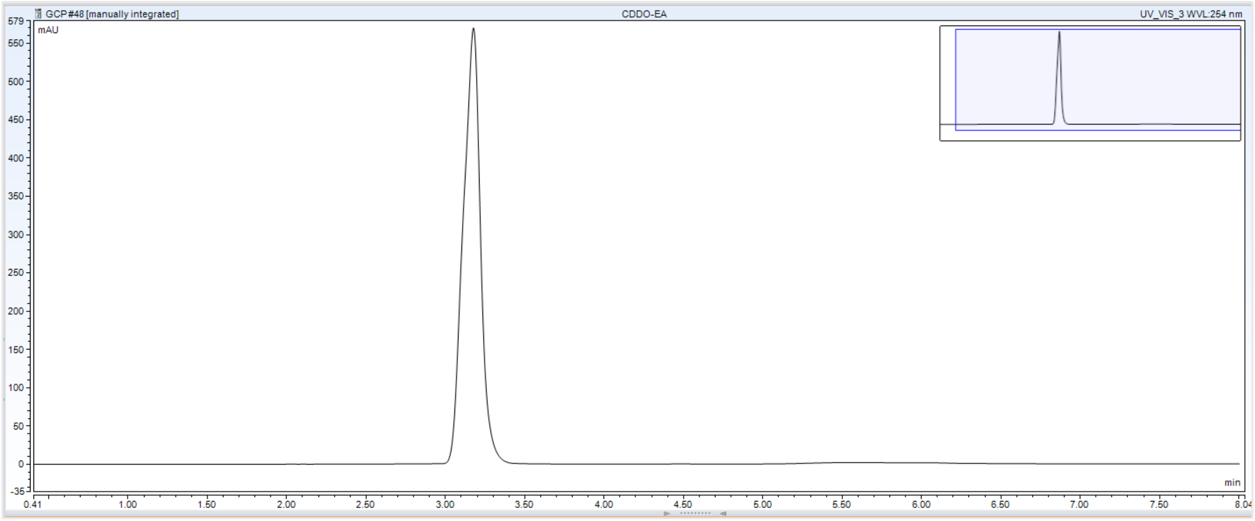

Figure S6. HPLC chromatogram of purified CDDO-EA: Flow rate 1.0 mL/min with MeOH.

HPLC was performed using a ThermoFisher Vanquish with an Acclaim 120 C18 (5 µM, 4.6 x 100mm) column. The purity of the synthesized CDDO-EA was > 99% .

Bibliography

1. L. Fu, G. W. Gribble., *Org. Lett.,* 2013, 15, 1622.
2. T. Honda, B. V. Rounds, L. Bore, H. J. Finlay, F. G. Favaloro, Jr., N. Suh, Y. Wang, M. B. Sporn, G. W. Gribble, *J. Med. Chem.* 2000, 43, 4233.
3. T. Honda, T. Janosik, Y. Honda, J. Han, K. T. Liby, C. R. Williams, R.D. Couch, A. C. Anderson, M. B. Sporn, G.W. Gribble, *J. Med. Chem.* 2004, 47, 4923.
